# Supplementary material for: Allelic Variation in Outer Membrane Protein A and Its Influence on Attachment of Escherichia coli to Corn Stover
Source: Front Microbiol. 2017 May 3;8:708. doi: 10.3389/fmicb.2017.00708 (PMC5413513; doi:10.3389/fmicb.2017.00708)
Supplement: Supplementary file 1 [file Table1.PDF]

Table S1: Primers

ompA sequencing

CACTGGCTGGTTTCGCTAC
GCGGCTGAGTTACAACGTCT

fliC gene knockout and confirmation

GGTGGAAACCCAATACGTAATCAACGACTTGCAATATAGGATAACGAATCGTGTAGGCTGGAGCTGCTTC
ATCAGGCAATTTGGCGTTGCCGTCAGTCTCAGTTAATCAGGTTACAACGAATGGGAATTAGCCATGGTCC
ATACTTGCCATGCGATTTCC
TACACCGTTTTCCATGAGCA

ompA gene knockout and confirmation

CTCGTTGGAGATATTCATGGCGTATTTTGGATGATAACGAGGCGCAAAAAGTGTAGGCTGGAGCTGCTTC
AAAGGCAAAAAAAACCCCGCAGCAGCGGGGTTTTTCTACCAGACGAGAACATGGGAATTAGCCATGGTCC
CCCCGGTGAAGGATTTAAC
AGGCATTTCAGTCAGTTGCTC

ompA cloning into pGEN-MCS

ATCGAAGCTTAGGCTTGTCTGAAGCGGTTT
CAGTGTCATGGTACTGGGACCAGCCCAGTTTAGCACCAGT
ACTGGTGCTAAACTGGGCTGGTCCCAGTACCATGACACTG
ATCGGCGGCCGCttaAGCCTGCGGCTGAGTTA

ompA cloning confirmation

CAAAAGAGATGGCCGATTTT
CAGCCCAGTTTAGCACCAGT
CCCCGGTGAAGGATTTAAC
CTTTTCGTTGGGATCTTTCG
