# Supplementary material for: Allelic Variation in Outer Membrane Protein A and Its Influence on Attachment of Escherichia coli to Corn Stover
Source: Front Microbiol. 2017 May 3;8:708. doi: 10.3389/fmicb.2017.00708 (PMC5413513; doi:10.3389/fmicb.2017.00708)
Supplement: Supplementary file 2 [file Table2.PDF]

Table S2: Amino acid sequence of OmpA from E. coli K12 strain MG1655 and each of the 78 isolates in this study.

>MG1655
MKKTAIAIAVALAGFATVAQAAPKDNTWYTGAKLGWSQYHDTGFINNNGPTHENQLGAGAFG
GYQVNPYVGFEMGYDWLGRMPYKGSVENGAYKAQGVQLTAKLGYPITDDLDIYTRLGGMVW
RADTKSNVYGKNHDTGVSPVFAGGVEYAITPEIATRLEYQWTNNIGDAHTIGTRPDNGMLSLGV
SYRFGQGEAAPVVAPAPAPAPEVQTKHFTLKSDVLFNFNKATLKPEGQAALDQLYSQLSNLDPK
DGSVVVLGYTDRIGSDAYNQGLSERRAQSVVDYLISKGIPADKISARGMGESNPVTGNTCDNVK
QRAALIDCLAPDRRVEIEVKGIKDVVTQPQA

>seq3
MKKTAIAIAVALAGFATVAQAAPKDNTWYTGAKLGWSQYHDTGFINNNGPTHENQLGAGAFG
GYQVNPYVGFEMGYDWLGRMPYKGSVVNGAYKAQGVQLTAKLGYPITDDLDIYTRLGGMVW
RADTKSNVYGKNHDTGVSPVFAGGVEYAITPEIATRLEYQWTNNIGDAHTIGTRPDNGMLSLGV
SYRFGQGEAAPVVAPAPAPAPEVQTKHFTLKSDVLFNFNKATLKPEGQAALDQLYSQLSNLDPK
DGSVVVLGYTDRIGSDAYNQGLSERRAQSVVDYLISKGIPADKISARGMGESNPVTGNTCDNVK
QRAALIDCLAPDRRVEIEVKGIKDVVTQPQA

>seq6
MKKTAIAIAVALAGFATVAQAAPKDNTWYTGAKLGWSQYHDTGFIPNNGPTHENQLGAGAFG
GYQVNPYVGFEMGYDWLGRMPYKGDNINGAYKAQGVQLTAKLGYPITDDLDIYTRLGGMVW
RADTKANVPGGASFKDHDTGVSPVFAGGVEYAITPEIATRLEYQWTNNIGDAHTIGTRPDNGM
LSLGVSYRFGQGEAAPVVAPAPAPAPEVQTKHFTLKSDVLFTFNKATLKPEGQAALDQLYSQLS
NLDPKDGSVVVLGYTDRIGSDAYNQGLSERRAQSVVDYLISKGIPADKISARGMGESNPVTGNT
CDNVKQRAALIDCLAPDRRVEIEVKGIKDVVTQPQA

>seq8
MKKTAIAIAVALAGFATVAQAAPKDNTWYTGAKLGWSQYHDTGFIPNNGPTHENQLGAGAFG
GYQVNPYVGFEMGYDWLGRMPYKGDNINGAYKAQGVQLTAKLGYPITDDLDVYTRLGGMVW
RADTKANVPGGASFKDHDTGVSPVFAGGVEYAITPEIATRLEYQWTNNIGDANTIGTRPDNGLL
SLGVSYRFGQGEAAPVVAPAPAPAPEVQTKHFTLKSDVLFNFNKATLKPEGQAALDQLYSQLSN
LDPKDGSVVVLGYTDRIGSDAYNQGLSERRAQSVVDYLISKGIPADKISARGMGESNPVTGNTC
DNVKQRAALIDCLAPDRRVEIEVKGIKDVVTQPQA

>seq9
MKKTAIAIAVALAGFATVAQAAPKDNTWYTGAKLGWSQYHDTGFINNNGPTHENQLGAGAFG
GYQVNPYVGFEMGYDWLGRMPYKGSVENGAYKAQGVQLTAKLGYPITDDLDIYTRLGGMVW
RADTKSNVYGKNHDTGVSPVFAGGVEYAITPEIATRLEYQWTNNIGDAHTIGTRPDNGMLSLGV
SYRFGQGEAAPVVAPAPAPAPEVQTKHFTLKSDVLFNFNKATLKPEGQAALDQLYSQLSNLDPK
DGSVVVLGYTDRIGSDAYNQGLSERRAQSVVDYLISKGIPADKISARGMGESNPVTGNTCDNVK
QRAALIDCLAPDRRVEIEVKGIKDVVTQPQA

>seq13
MKKTAIAIAVALAGFATVAQAAPKDNTWYTGAKLGWSQYHDTGFIPNNGPTHENQLGAGAFG
GYQVNPYVGFEMGYDWLGRMPYKGDNINGAYKAQGVQLTAKLGYPITDDLDVYTRLGGMVW
RADTKANVPGGASFKDHDTGVSPVFAGGVEYAITPEIATRLEYQWTNNIGDANTIGTRPDNGLL
SLGVSYRFGQGEAAPVVAPAPAPAPEVQTKHFTLKSDVLFNFNKATLKPEGQAALDQLYSQLSN
LDPKDGSVVVLGYTDRIGSDAYNQGLSERRAQSVVDYLISKGIPADKISARGMGESNPVTGNTC
DNVKQRAALIDCLAPDRRVEIEVKGIKDVVTQPQA

>seq16
MKKTAIAIAVALAGFATVAQAAPKDNTWYTGAKLGWSQYHDTGFI**D**NNGPTHENQLGAGAFG
GYQVNPYVGFEMGYDWLGRMPYKG**SVE**NGAYKAQGVQLTAKLGYPITDDLD**V**YTRLGGMV
WRADTK**SNVYG**K**N**HDTGVSPVFAGGVE**Y**AITPEIATRLEYQWTNNIGDA**H**TIGTRPDNG**M**LSL
GVSYRFGQGE**V**APVV**A**PAPAPAPEVQTKHFTLKSDVLF**T**FNKATLKPEGQAALDQLYSQLSNLD
PKDGSVVVLGYTDRIGSDAYNQ**A**LSERRAQSVVDYLISKGIP**A**DKISARGMGESNPVTGNTCDN
VKQRAALIDCLAPDRRVEIEVKGIKDVVTQPQA
>seq18
MKKTAIAIAVALAGFATVAQAAPKDNTWYTGAKLGWSQYHDTGFI**P**NNGPTHENQLGAGAFG
GYQVNPYVGFEMGYDWLGRMPYKG**DNI**NGAYKAQGVQLTAKLGYPITDDLD**V**YTRLGGMVW
RADTK**ANVPGGASF**K**D**HDTGVSPVFAGGVE**Y**AITPEIATRLEYQWTNNIGDA**N**TIGTRPDNG**L**L
SLGVSYRFGQGE**A**APVV**T**PAPAPAPEVQTKHFTLKSDVLF**N**FNKATLKPEGQAALDQLYSQLSN
LDPKDGSVVVLGYTDRIGSDAYNQ**G**LSERRAQSVVDYLISKGIP**A**DKISARGMGESNPVTGNTC
DNVKQRAALIDCLAPDRRVEIEVKGIKDVVTQPQA
>seq26
MKKTAIAIAVALAGFATVAQAAPKDNTWYTGAKLGWSQYHDTGFI**N**NNGPTHENQLGAGAFG
GYQVNPYVGFEMGYDWLGRMPYKG**SVE**NGAYKAQGVQLTAKLGYPITDDLD**V**YTRLGGMV
WRADTK**SNVYG**K**N**HDTGVSPVFAGGVE**Y**AITPEIATRLEYQWTNNIGDA**H**TIGTRPDNG**M**LSL
GVSYRFGQGE**A**APVV**A**PAPAPAPEVQTKHFTLKSDVLF**T**FNKATLKPEGQAALDQLYSQLSNLD
PKDGSVVVLGYTDRIGSDAYNQ**G**LSERRAQSVVDYLISKGIP**A**DKISARGMGESNPVTGNTCDN
VKQRAALIDCLAPDRRVEIEVKGIKDVVTQPQA

>seq28
MKKTAIAIAVALAGFATVAQAAPKDNTWYTGAKLGWSQYHDTGFI**N**NNGPTHENQLGAGAFG
GYQVNPYVGFEMGYDWLGRMPYKG**SVE**NGAYKAQGVQLTAKLGYPITDDLD**I**YTRLGGMVW
RADTK**SNVYG**K**N**HDTGVSPVFAGGVE**Y**AITPEIATRLEYQWTNNIGDA**H**TIGTRPDNG**M**LSLGV
SYRFGQGE**A**APVV**A**PAPAPAPEVQTKHFTLKSDVLF**N**FNKATLKPEGQAALDQLYSQLSNLDPK
DGSVVVLGYTDRIGSDAYNQ**G**LSERRAQSVVDYLISKGIP**A**DKISARGMGESNPVTGNTCDNVK
QRAALIDCLAPDRRVEIEVKGIKDVVTQPQA

>seq32
MKKTAIAIAVALAGFATVAQAAPKDNTWYTGAKLGWSQYHDTGFI**D**NNGPTHENQLGAGAFG
GYQVNPYVGFEMGYDWLGRMPYKG**DNI**NGAYKAQGVQLTAKLGYPITDDLD**I**YTRLGGMVW
RADTK**ANVPGGASF**K**D**HDTGVSPVFAGGVE**Y**AITPEIATRLEYQWTNNIGDA**H**TIGTRPDNG**L**L
SLGVSYRFGQGE**A**APVV**A**PAPAPAPEVQTKHFTLKSDVLF**N**FNKATLKPEGQAALDQLYSQLSN
LDPKDGSVVVLGYTDRIGSDAYNQ**G**LSERRAQSVVDYLISKGIP**A**DKISARGMGESNPVTGNTC
DNVKQRAALIDCLAPDRRVEIEVKGIKDVVTQPQA

>seq36
MKKTAIAIAVALAGFATVAQAAPKDNTWYTGAKLGWSQYHDTGFI**N**NNGPTHENQLGAGAFG
GYQVNPYVGFEMGYDWLGRMPYKG**SVE**NGAYKAQGVQLTAKLGYPITDDLD**V**YTRLGGMV
WRADTK**SNFDG**K**N**HDTGVSPVFAGGVE**Y**AITPEIATRLEYQWTNNIGDA**H**TIGTRPDNG**M**LSL
GVSYRFGQGE**A**APVV**A**PAPAPAPEVQTKHFTLKSDVLF**T**FNKATLKPEGQAALDQLYSQLSNLD
PKDGSVVVLGYTDRIGSDAYNQ**A**LSERRAQSVVDYLISKGIP**A**DKISARGMGESNPVTGNTCDN
VKQRAALIDCLAPDRRVEIEVKGIKDVVTQPQA

>seq44
MKKTAIAIAVALAGFATVAQAAPKDNTWYTGAKLGWSQYHDTGFI**D**NNGPTHENQLGAGAFG
GYQVNPYVGFEMGYDWLGRMPYKG**DNI**NGAYKAQGVQLTAKLGYPITDDLD**V**YTRLGGMVW
RADTK**SNVYG**K**N**HDTGVSPVFAGGVE**Y**AITPEIATRLEYQWTNNIGDA**H**TIGTRPDNG**L**LSLGV
SYRFGQGE**A**APVV**A**PAPAPAPEVQTKHFTLKSDVLF**N**FNKATLKPEGQAALDQLYSQLSNLDPK

DGSVVVLGYTDRIGSDAYNQ**G**LSERRAQSVVDYLISKGIP**A**DKISARGMGESNPVTGNTCDNVK
QRAALIDCLAPDRRVEIEVKGIKDVVTQPQA

>seq48
MKKTAIAIAVALAGFATVAQAAPKDNTWYTGAKLGWSQYHDTGFI**N**NNGPTHENQLGAGAFG
GYQVNPYVGFEMGYDWLGRMPYKG**SVE**NGAYKAQGVQLTAKLGYPITDDLD**I**YTRLGGMVW
RADTK**SNVYG**K**N**HDTGVSPVFAGGVE**Y**AITPEIATRLEYQWTNNIGDA**H**TIGTRPDNG**M**LSLGV
SYRFGQGE**A**APVV**A**PAPAPAPEVQTKHFTLKSDVLF**N**FNKATLKPEGQAALDQLYSQLSNLDPK
DGSVVVLGYTDRIGSDAYNQ**G**LSERRAQSVVDYLISKGIP**A**DKISARGMGESNPVTGNTCDNVK
QRAALIDCLAPDRRVEIEVKGIKDVVTQPQA

>seq53
MKKTAIAIAVALAGFATVAQAAPKDNTWYTGAKLGWSQYHDTGFI**P**NNGPTHENQLGAGAFG
GYQVNPYVGFEMGYDWLGRMPYKG**DNI**NGAYKAQGVQLTAKLGYPITDDLD**I**YTRLGGMVW
RADTK**ANVPGGASF**K**D**HDTGVSPVFAGGVE**Y**AITPEIATRLEYQWTNNIGDA**H**TIGTRPDNG**M**
LSLGVSYRFGQGE**V**APVV**A**PAPAPAPEVQTKHFTLKSDVLF**T**FNKATLKPEGQAALDQLYSQLS
NLDPKDGSVVVLGYTDRIGSDAYNQ**A**LSERRAQSVVDYLISKGIP**A**DKISARGMGESNPVTGNT
CDNVKQRAALIDCLAPDRRVEIEVKGIKDVVTQPQA

>seq57
MKKTAIAIAVALAGFATVAQAAPKDNTWYTGAKLGWSQYHDTGFI**P**NNGPTHENQLGAGAFG
GYQVNPYVGFEMGYDWLGRMPYKG**DNI**NGAYKAQGVQLTAKLGYPITDDLD**I**YTRLGGMVW
RADTK**ANVPGGASF**K**D**HDTGVSPVFAGGVE**Y**AITPEIATRLEYQWTNNIGDA**N**TIGTRPDNG**L**L
SLGVSYRFGQGE**A**APVV**A**PAPAPAPEVQTKHFTLKSDVLF**N**FNKATLKPEGQAALDQLYSQLSN
LDPKDGSVVVLGYTDRIGSDAYNQ**G**LSERRAQSVVDYLISKGIP**A**DKISARGMGESNPVTGNTC
DNVKQRAALIDCLAPDRRVEIEVKGIKDVVTQPQA

>seq60
MKKTAIAIAVALAGFATVAQAAPKDNTWYTGAKLGWSQYHDTGFI**N**NNGPTHENQLGAGAFG
GYQVNPYVGFEMGYDWLGRMPYKG**SVE**NGAYKAQGVQLTAKLGYPITDDLD**I**YTRLGGMVW
RADTK**SNVYG**K**N**HDTGVSPVFAGGVE**Y**AITPEIATRLEYQWTNNIGDA**H**TIGTRPDNG**M**LSLGV
SYRFGQGE**A**APVV**A**PAPAPAPEVQTKHFTLKSDVLF**N**FNKATLKPEGQAALDQLYSQLSNLDPK
DGSVVVLGYTDRIGSDAYNQ**G**LSERRAQSVVDYLISKGIP**A**DKISARGMGESNPVTGNTCDNVK
QRAALIDCLAPDRRVEIEVKGIKDVVTQPQA

>seq62
MKKTAIAIAVALAGFATVAQAAPKDNTWYTGAKLGWSQYHDTGFI**D**NNGPTHENQLGAGAFG
GYQVNPYVGFEMGYDWLGRMPYKG**SVE**NGAYKAQGVQLTAKLGYPITDDLD**V**YTRLGGMV
WRADTK**AHNNVTGESE**K**N**HDTGVSPVFAGGVE**W**AITPEIATRLEYQWTNNIGDA**H**TIGTRPDN
G**L**LSLGVSYRFGQGE**A**APVV**A**PAPAPAPEVQTKHFTLKSDVLF**N**FNKATLKPEGQAALDQLYSQ
LSNLDPKDGSVVVLGYTDRIGSDAYNQ**G**LSERRAQSVVDYLISKGIP**A**DKISARGMGESNPVTG
NTCDNVKQRAALIDCLAPDRRVEIEVKGIKDVVTQPQA

>seq69
MKKTAIAIAVALAGFATVAQAAPKDNTWYTGAKLGWSQYHDTGFI**N**NNGPTHENQLGAGAFG
GYQVNPYVGFEMGYDWLGRMPYKG**SVE**NGAYKAQGVQLTAKLGYPITDDLD**I**YTRLGGMVW
RADTK**SNVYG**K**N**HDTGVSPVFAGGVE**Y**AITPEIATRLEYQWTNNIGDA**H**TIGTRPDNG**M**LSLGV
SYRFGQGE**A**APVV**A**PAPAPAPEVQTKHFTLKSDVLF**N**FNKATLKPEGQAALDQLYSQLSNLDPK
DGSVVVLGYTDRIGSDAYNQ**G**LSERRAQSVVDYLISKGIP**A**DKISARGMGESNPVTGNTCDNVK
QRAALIDCLAPDRRVEIEVKGIKDVVTQPQA

>seq83
MKKTAIAIAVALAGFATVAQAAPKDNTWYTGAKLGWSQYHDTGFI**P**NNGPTHENQLGAGAFG
GYQVNPYVGFEMGYDWLGRMPYKG**DNI**NGAYKAQGVQLTAKLGYPITDDLD**I**YTRLGGMVW
RADTK**ANVPGGASF**K**D**HDTGVSPVFAGGVE**Y**AITPEIATRLEYQWTNNIGDA**N**TIGTRPDNG**L**L
SLGVSYRFGQGE**A**APVV**A**PAPAPAPEVQTKHFTLKSDVLF**N**FNKATLKPEGQAALDQLYSQLSN
LDPKDGSVVVLGYTDRIGSDAYNQ**G**LSERRAQSVVDYLISKGIP**A**DKISARGMGESNPVTGNTC
DNVKQRAALIDCLAPDRRVEIEVKGIKDVVTQPQA

>seq84
MKKTAIAIAVALAGFATVAQAAPKDNTWYTGAKLGWSQYHDTGFI**N**NNGPTHENQLGAGAFG
GYQVNPYVGFEMGYDWLGRMPYKG**SVE**NGAYKAQGVQLTAKLGYPITDDLD**I**YTRLGGMVW
RADTK**SNVYG**K**N**HDTGVSPVFAGGVE**Y**AITPEIATRLEYQWTNNIGDA**H**TIGTRPDNG**M**LSLGV
SYRFGQGE**A**APVV**A**PAPAPAPEVQTKHFTLKSDVLF**N**FNKATLKPEGQAALDQLYSQLSNLDPK
DGSVVVLGYTDRIGSDAYNQ**G**LSERRAQSVVDYLISKGIP**A**DKISARGMGESNPVTGNTCDNVK
QRAALIDCLAPDRRVEIEVKGIKDVVTQPQA

>seq89
MKKTAIAIAVALAGFATVAQAAPKDNTWYTGAKLGWSQYHDTGFI**P**NNGPTHENQLGAGAFG
GYQVNPYVGFEMGYDWLGRMPYKG**DNI**NGAYKAQGVQLTAKLGYPITDDLD**V**YTRLGGMVW
RADTK**ANVPGGASF**K**D**HDTGVSPVFAGGVE**Y**AITPEIATRLEYQWTNNIGDA**N**TIGTRPDNG**L**L
SLGVSYRFGQGE**A**APVV**A**PAPAPAPEVQTKHFTLKSDVLF**N**FNKATLKPEGQAALDQLYSQLSN
LDPKDGSVVVLGYTDRIGSDAYNQ**G**LSERRAQSVVDYLISKGIP**A**DKISARGMGESNPVTGNTC
DNVKQRAALIDCLAPDRRVEIEVKGIKDVVTQPQA

>seq98
MKKTAIAIAVALAGFATVAQAAPKDNTWYTGAKLGWSQYHDTGFI**P**NNGPTHENQLGAGAFG
GYQVNPYVGFEMGYDWLGRMPYKG**DNI**NGAYKAQGVQLTAKLGYPITDDLD**V**YTRLGGMVW
RADTK**ANVPGGASF**K**D**HDTGVSPVFAGGVE**Y**AITPEIATRLEYQWTNNIGDA**H**TIGTRPDNG**M**
LSLGVSYRFGQGE**A**APVV**A**PAPAPAPEVQTKHFTLKSDVLF**N**FNKATLKPEGQAALDQLYSQLS
NLDPKDGSVVVLGYTDRIGSDAYNQ**G**LSERRAQSVVDYLISKGIP**A**DKISARGMGESNPVTGNT
CDNVKQRAALIDCLAPDRRVEIEVKGIKDVVTQPQA

>seq100
MKKTAIAIAVALAGFATVAQAAPKDNTWYTGAKLGWSQYHDTGFI**P**NNGPTHENQLGAGAFG
GYQVNPYVGFEMGYDWLGRMPYKG**DNI**NGAYKAQGVQLTAKLGYPITDDLD**V**YTRLGGMVW
RADTK**ANVPGVASF**K**D**HDTGVSPVFAGGVE**Y**AITPEIATRLEYQWTNNIGDA**N**TIGTRPDNG**L**L
SLGVSYRFGQGE**A**APVV**A**PAPAPAPEVQTKHFTLKSDVLF**N**FNKATLKPEGQAALDQLYSQLSN
LDPKDGSVVVLGYTDRIGSDAYNQ**G**LSERRAQSVVDYLISKGIP**A**DKISARGMGESNPVTGNTC
DNVKQRAALIDCLAPDRRVEIEVKGIKDVVTQPQA

>seq105
MKKTAIAIAVALAGFATVAQAAPKDNTWYTGAKLGWSQYHDTGFI**N**NNGPTHENQLGAGAFG
GYQVNPYVGFEMGYDWLGRMPYKG**SVA**NGAYKAQGVQLTAKLGYPITDDLD**I**YTRLGGMVW
RADTK**SNVYG**K**N**HDTGVSPVFAGGVE**Y**AITPEIATRLEYQWTNNIGDA**H**TIGTRPDNG**M**LSLGV
SYRFGQGE**A**APVV**A**PAPAPAPEVQTKHFTLKSDVLF**N**FNKATLKPEGQAALDQLYSQLSNLDPK
DGSVVVLGYTDRIGSDAYNQ**G**LSERRAQSVVDYLISKGIP**A**DKISARGMGESNPVTGNTCDNVK
QRAALIDCLAPDRRVEIEVKGIKDVVTQPQA

>seq108
MKKTAIAIAVALAGFATVAQAAPKDNTWYTGAKLGWSQYHDTGFI**N**NNGPTHENQLGAGAFG
GYQVNPYVGFEMGYDWLGRMPYKG**SVE**NGAYKAQGVQLTAKLGYPITDDLD**I**YTRLGGMVW

RADTK**SNVYG**K**N**HDTGVSPVFAGGVE**Y**AITPEIATRLEYQWTNNIGDA**H**TIGTRPDNG**M**LSLGV
SYRFGQGE**A**APVV**A**PAPAPAPEVQTKHFTLKSDVLF**N**FNKATLKPEGQAALDQLYSQLSNLDPK
DGSVVVLGYTDRIGSDAYNQ**G**LSERRAQSVVDYLISKGIP**A**DKISARGMGESNPVTGNTCDNVK
QRAALIDCLAPDRRVEIEVKGIKDVVTQPQA

>seq110
MKKTAIAIAVALAGFATVAQAAPKDNTWYTGAKLGWSQYHDTGFI**N**NNGPTHENQLGAGAFG
GYQVNPYVGFEMGYDWLGRMPYKG**DNI**NGAYKAQGVQLTAKLGYPITDDLD**I**YTRLGGMVW
RADTK**ANVPGGASF**K**D**HDTGVSPVFAGGVE**Y**AITPEIATRLEYQWTNNIGDA**H**TIGTRPDNG**L**L
SLGVSYRFGQGE**A**APVV**A**PAPAPAPEVQTKHFTLKSDVLF**N**FNKATLKPEGQAALDQLYSQLSN
LDPKDGSVVVLGYTDRIGSDAYNQ**G**LSERRAQSVVDYLISKGIP**A**DKISARGMGESNPVTGNTC
DNVKQRAALIDCLAPDRRVEIEVKGIKDVVTQPQA

>seq111
MKKTAIAIAVALAGFATVAQAAPKDNTWYTGAKLGWSQYHDTGFI**N**NNGPTHENQLGAGAFG
GYQVNPYVGFEMGYDWLGRMPYKG**SVE**NGAYKAQGVQLTAKLGYPITDDLD**V**YTRLGGMV
WRADTK**SNFDG**K**N**HDTGVSPVFAGGVE**Y**AITPEIATRLEYQWTNNIGDA**H**TIGTRPDNG**M**LSL
GVSYRFGQGE**A**APVV**A**PAPAPAPEVQTKHFTLKSDVLF**T**FNKATLKPEGQAALDQLYSQLSNLD
PKDGSVVVLGYTDRIGSDAYNQ**G**LSERRAQSVVDYLISKGIP**A**DKISARGMGESNPVTGNTCDN
VKQRAALIDCLAPDRRVEIEVKGIKDVVTQPQA

>seq117
MKKTAIAIAVALAGFATVAQAAPKDNTWYTGAKLGWSQYHDTGFI**P**NNGPTHENQLGAGAFG
GYQVNPYVGFEMGYDWLGRMPYKG**DNI**NGAYKAQGVQLTAKLGYPITDDLD**I**YTRLGGMVW
RADTK**ANVPGGASF**K**D**HDTGVSPVFAGGVE**Y**AITPEIATRLEYQWTNNIGDA**H**TIGTRPDNG**M**
LSLGVSYRFGQGE**A**APVV**A**PAPAPAPEVQTKHFTLKSDVLF**T**FNKATLKPEGQAALDQLYSQLS
NLDPKDGSVVVLGYTDRIGSDAYNQ**G**LSERRAQSVVDYLISKGIP**A**DKISARGMGESNPVTGNT
CDNVKQRAALIDCLAPDRRVEIEVKGIKDVVTQPQA

>seq122
MKKTAIAIAVALAGFATVAQAAPKDNTWYTGAKLGWSQYHDTGFI**N**NNGPTHENQLGAGAFG
GYQVNPYVGFEMGYDWLGRMPYKG**SVE**NGAYKAQGVQLTAKLGYPITDDLD**V**YTRLGGMV
WRADTK**SNFDG**K**N**HDTGVSPVFAGGVE**Y**AITPEIATRLEYQWTNNIGDA**H**TIGTRPDNG**M**LSL
GVSYRFGQGE**A**APVV**A**PAPAPAPEVQTKHFTLKSDVLF**T**FNKATLKPEGQAALDQLYSQLSNLD
PKDGSVVVLGYTDRIGSDAYNQ**A**LSERRAQSVVDYLISKGIP**A**DKISARGMGESNPVTGNTCDN
VKQRAALIDCLAPDRRVEIEVKGIKDVVTQPQA

>seq127
MKKTAIAIAVALAGFATVAQAAPKDNTWYTGAKLGWSQYHDTGFI**D**NNGPTHENQLGAGAFG
GYQVNPYVGFEMGYDWLGRMPYKG**SVE**NGAYKAQGVQLTAKLGYPITDDLD**V**YTRLGGMV
WRADTK**SNFDG**K**N**HDTGVSPVFAGGVE**Y**AITPEIATRLEYQWTNNIGDA**H**TIGTRPDNG**M**LSL
GVSYRFGQGE**A**APVV**A**PAPAPAPEVQTKHFTLKSDVLF**T**FNKATLKPEGQAALDQLYSQLSNLD
PKDGSVVVLGYTDRIGSDAYNQ**G**LSERRAQSVVDYLISKGIP**A**DKISARGMGESNPVTGNTCDN
VKQRAALIDCLAPDRRVEIEVKGIKDVVTQPQA

>seq128
MKKTAIAIAVALAGFATVAQAAPKDNTWYTGAKLGWSQYHDTGFI**N**NNGPTHENQLGAGAFG
GYQVNPYVGFEMGYDWLGRMPYKG**SVE**NGAYKAQGVQLTAKLGYPITDDLD**V**YTRLGGMV
WRADTK**SNFDG**K**N**HDTGVSPVFAGGVE**Y**AITPEIATRLEYQWTNNIGDA**H**TIGTRPDNG**M**LSL
GVSYRFGQGE**A**APVV**A**PAPAPAPEVQTKHFTLKSDVLF**T**FNKATLKPEGQAALDQLYSQLSNLD

PKDGSVVVLGYTDRIGSDAYNQ**G**LSERRAQSVVDYLISKGIP**A**DKISARGMGESNPVTGNTCDN
VKQRAALIDCLAPDRRVEIEVKGIKDVVTQPQA

>seq130
MKKTAIAIAVALAGFATVAQAAPKDNTWYTGAKLGWSQYHDTGFI**N**NNGPTHENQLGAGAFG
GYQVNPYVGFEMGYDWLGRMPYKG**SVE**NGAYKAQGVQLTAKLGYPITDDLD**I**YTRLGGMVW
RADTK**SNVYG**K**N**HDTGVSPVFAGGVE**Y**AITPEIATRLEYQWTNNIGDA**H**TIGTRPDNG**M**LSLGV
SYRFGQGE**A**APVV**A**PAPAPAPEVQTKHFTLKSDVLF**N**FNKATLKPEGQAALDQLYSQLSNLDPK
DGSVVVLGYTDRIGSDAYNQ**G**LSERRAQSVVDYLISKGIP**A**DKISARGMGESNPVTGNTCDNVK
QRAALIDCLAPDRRVEIEVKGIKDVVTQPQA

>seq141
MKKTAIAIAVALAGFATVAQAAPKDNTWYTGAKLGWSQYHDTGFI**N**NNGPTHENQLGAGAFG
GYQVNPYVGFEMGYDWLGRMPYKG**SVE**NGAYKAQGVQLTAKLGYPITDDLD**V**YTRLGGMV
WRADTK**SNFDG**K**N**HDTGVSPVFAGGVE**Y**AITPEIATRLEYQWTNNIGDA**H**TIGTRPDNG**M**LSL
GVSYRFGQGE**A**APVV**A**PAPAPAPEVQTKHFTLKSDVLF**T**FNKATLKPEGQAALDQLYSQLSNLD
PKDGSVVVLGYTDRIGSDAYNQ**G**LSERRAQSVVDYLISKGIP**A**DKISARGMGESNPVTGNTCDN
VKQRAALIDCLAPDRRVEIEVKGIKDVVTQPQA

>seq144
MKKTAIAIAVALAGFATVAQAAPKDNTWYTGAKLGWSQYHDTGFI**D**NNGPTHENQLGAGAFG
GYQVNPYVGFEMGYDWLGRMPYKG**SVE**NGAYKAQGVQLTAKLGYPITDDLD**V**YTRLGGMV
WRADTK**AHNNVTGESE**K**N**HDTGVSPVFAGGVE**W**AITPEIATRLEYQWTNNIGDA**H**TIGTRPDN
G**L**LSLGVSYRFGQGE**A**APVV**A**PAPAPAPEVQTKHFTLKSDVLF**N**FNKATLKPEGQAALDQLYSQ
LSNLDPKDGSVVVLGYTDRIGSDAYNQ**G**LSERRAQSVVDYLISKGIP**A**DKISARGMGESNPVTG
NTCDNVKQRAALIDCLAPDRRVEIEVKGIKDVVTQPQA

>seq146
MKKTAIAIAVALAGFATVAQAAPKDNTWYTGAKLGWSQYHDTGFI**P**NNGPTHENQLGAGAFG
GYQVNPYVGFEMGYDWLGRMPYKG**DNI**NGAYKAQGVQLTAKLGYPITDDLD**I**YTRLGGMVW
RADTK**ANVPGGASF**K**D**HDTGVSPVFAGGVE**Y**AITPEIATRLEYQWTNNIGDA**H**TIGTRPDNG**M**
LSLGVSYRFGQGE**A**APVV**A**PAPAPAPEVQTKHFTLKSDVLF**T**FNKATLKPEGQAALDQLYSQLS
NLDPKDGSVVVLGYTDRIGSDAYNQ**G**LSERRAQSVVDYLISKGIP**A**DKISARGMGESNPVTGNT
CDNVKQRAALIDCLAPDRRVEIEVKGIKDVVTQPQA

>seq147
MKKTAIAIAVALAGFATVAQAAPKDNTWYTGAKLGWSQYHDTGFI**N**NNGPTHENQLGAGAFG
GYQVNPYVGFEMGYDWLGRMPYKG**SVE**NGAYKAQGVQLTAKLGYPITDDLD**I**YTRLGGMVW
RADTK**SNVYG**K**N**HDTGVSPVFAGGVE**Y**AITPEIATRLEYQWTNNIGDA**H**TIGTRPDNG**M**LSLGV
SYRFGQGE**A**APVV**A**PAPAPAPEVQTKHFTLKSDVLF**N**FNKATLKPEGQAALDQLYSQLSNLDPK
DGSVVVLGYTDRIGSDAYNQ**G**LSERRAQSVVDYLISKGIP**A**DKISARGMGESNPVTGNTCDNVK
QRAALIDCLAPDRRVEIEVKGIKDVVTQPQA

>seq148
MKKTAIAIAVALAGFATVAQAAPKDNTWYTGAKLGWSQYHDTGFI**P**NNGPTHENQLGAGAFG
GYQVNPYVGFEMGYDWLGRMPYKG**DNI**NGAYKAQGVQLTAKLGYPITDDLD**V**YTRLGGMVW
RADTK**ANVPGGASF**K**D**HDTGVSPVFAGGVE**Y**AITPEIATRLEYQWTNNIGDA**N**TIGTRPDNG**L**L
SLGVSYRFGQGE**A**APVV**A**PAPAPAPEVQTKHFTLKSDVLF**N**FNKATLKPEGQAALDQLYSQLSN
LDPKDGSVVVLGYTDRIGSDAYNQ**G**LSERRAQSVVDYLISKGIP**A**DKISARGMGESNPVTGNTC
DNVKQRAALIDCLAPDRRVEIEVKGIKDVVTQPQA

>seq157
MKKTAIAIAVALAGFATVAQAAPKDNTWYTGAKLGWSQYHDTGFI**N**NNGPTHENQLGAGAFG
GYQVNPYVGFEMGYDWLGRMPYKG**SVE**NGAYKAQGVQLTAKLGYPITDDLD**V**YTRLGGMV
WRADTK**SNVYG**K**N**HDTGVSPVFAGGVE**Y**AITPEIATRLEYQWTNNIGDA**H**TIGTRPDNG**M**LSL
GVSYRFGQGE**A**APVV**A**PAPAPAPEVQTKHFTLKSDVLF**T**FNKATLKPEGQAALDQLYSQLSNLD
PKDGSVVVLGYTDRIGSDAYNQ**A**LSERRAQSVVDYLISKGIP**A**DKISARGMGESNPVTGNTCDN
VKQRAALIDCLAPDRRVEIEVKGIKDVVTQPQA

>seq158
MKKTAIAIAVALAGFATVAQAAPKDNTWYTGAKLGWSQYHDTGFI**P**NNGPTHENQLGAGAFG
GYQVNPYVGFEMGYDWLGRMPYKG**DNI**NGAYKAQGVQLTAKLGYPITDDLD**V**YTRLGGMVW
RADTK**ANVPGGASF**K**D**HDTGVSPVFAGGVE**Y**AITPEIATRLEYQWTNNIGDA**H**TIGTRPDNG**M**
LSLGVSYRFGQGE**A**APVV**A**PAPAPAPEVQTKHFTLKSDVLF**N**FNKATLKPEGQAALDQLYSQLS
NLDPKDGSVVVLGYTDRIGSDAYNQ**G**LSERRAQSVVDYLISKGIP**A**DKISARGMGESNPVTGNT
CDNVKQRAALIDCLAPDRRVEIEVKGIKDVVTQPQA

>seq160
MKKTAIAIAVALAGFATVAQAAPKDNTWYTGAKLGWSQYHDTGFI**P**NNGPTHENQLGAGAFG
GYQVNPYVGFEMGYDWLGRMPYKG**DNI**NGAYKAQGVQLTAKLGYPITDDLD**I**YTRLGGMVW
RADTK**ANVPGGASF**K**D**HDTGVSPVFAGGVE**Y**AITPEIATRLEYQWTNNIGDA**H**TIGTRPDNG**M**
LSLGVSYRFGQGE**A**APVV**A**PAPAPAPEVQTKHFTLKSDVLF**T**FNKATLKPEGQAALDQLYSQLS
NLDPKDGSVVVLGYTDRIGSDAYNQ**A**LSERRAQSVVDYLISKGIP**A**DKISARGMGESNPVTGNT
CDNVKQRAALIDCLAPDRRVEIEVKGIKDVVTQPQA

>seq182
MKKTAIAIAVALAGFATVAQAAPKDNTWYTGAKLGWSQYHDTGFI**N**NNGPTHENQLGAGAFG
GYQVNPYVGFEMGYDWLGRMPYKG**SVE**NGAYKAQGVQLTAKLGYPITDDLD**V**YTRLGGMV
WRADTK**SNVYG**K**N**HDTGVSPVFAGGVE**Y**AITPEIATRLEYQWTNNIGDA**H**TIGTRPDNG**M**LSL
GVSYRFGQGE**A**APVV**A**PAPAPAPEVQTKHFTLKSDVLF**T**FNKATLKPEGQAALDQLYSQLSNLD
PKDGSVVVLGYTDRIGSDAYNQ**A**LSERRAQSVVDYLISKGIP**A**DKISARGMGESNPVTGNTCDN
VKQRAALIDCLAPDRRVEIEVKGIKDVVTQPQA

>seq184
MKKTAIAIAVALAGFATVAQAAPKDNTWYTGAKLGWSQYHDTGFI**P**NNGPTHENQLGAGAFG
GYQVNPYVGFEMGYDWLGRMPYKG**DNI**NGAYKAQGVQLTAKLGYPITDDLD**V**YTRLGGMVW
RADTK**ANVPGGASF**K**D**HDTGVSPVFAGGVE**Y**AITPEIATRLEYQWTNNIGDA**H**TIGTRPDNG**M**
LSLGVSYRFGQGE**A**APVV**A**PAPAPAPEVQTKHFTLKSDVLF**N**FNKATLKPEGQAALDQLYSQLS
NLDPKDGSVVVLGYTDRIGSDAYNQ**G**LSERRAQSVVDYLISKGIP**A**DKISARGMGESNPVTGNT
CDNVKQRAALIDCLAPDRRVEIEVKGIKDVVTQPQA

>seq185
MKKTAIAIAVALAGFATVAQAAPKDNTWYTGAKLGWSQYHDTGFI**N**NNGPTHENQLGAGAFG
GYQVNPYVGFEMGYDWLGRMPYKG**SVE**NGAYKAQGVQLTAKLGYPITDDLD**V**YTRLGGMV
WRADTK**SNFDG**K**N**HDTGVSPVFAGGVE**Y**AITPEIATRLEYQWTNNIGDA**H**TIGTRPDNG**M**LSL
GVSYRFGQGE**A**APVV**A**PAPAPAPEVQTKHFTLKSDVLF**T**FNKATLKPEGQAALDQLYSQLSNLD
PKDGSVVVLGYTDRIGSDAYNQ**G**LSERRAQSVVDYLISKGIP**A**DKISARGMGESNPVTGNTCDN
VKQRAALIDCLAPDRRVEIEVKGIKDVVTQPQA

>seq186
MKKTAIAIAVALAGFATVAQAAPKDNTWYTGAKLGWSQYHDTGFI**D**NNGPTHENQLGAGAFG
GYQVNPYVGFEMGYDWLGRMPYKG**SVE**NGAYKAQGVQLTAKLGYPITDDLD**V**YTRLGGMV

WRADTKSNVYGKNHDTGVSPVFAGGVEYAITPEIATRLEYQWTNNIGDAHTIGTRPDNGMLSL
GVSYRFGQGEVAPVVAPAPAPAPEVQTKHFTLKSDVLFTFNKATLKPEGQAALDQLYSQLSNLD
PKDGSVVVLGYTDRIGSDAYNQALSERRAQSVVDYLISKGIPADKISARGMGESNPVTGNTCDN
VKQRAALIDCLAPDRRVEIEVKGIKDVVTQPQA

>seq196
MKKTAIAIAVALAGFATVAQAAPKDNTWYTGAKLGWSQYHDTGFINNNGPTHENQLGAGAFG
GYQVNPYVGFEMGYDWLGRMPYKGSVENGAYKAQGVQLTAKLGYPITDDLDIYTRLGGMVW
RADTKSNVYGKNHDTGVSPVFAGGVEYAITPEIATRLEYQWTNNIGDAHTIGTRPDNGMLSLGV
SYRFGQGEAAPVVAPAPAPAPEVQTKHFTLKSDVLFNFNKATLKPEGQAALDQLYSQLSNLDPK
DGSVVVLGYTDRIGSDAYNQGLSERRAQSVVDYLISKGIPADKISARGMGESNPVTGNTCDNVK
QRAALIDCLAPDRRVEIEVKGIKDVVTQPQA

>seq201
MKKTAIAIAVALAGFATVAQAAPKDNTWYTGAKLGWSQYHDTGFINNNGPTHENQLGAGAFG
GYQVNPYVGFEMGYDWLGRMPYKGSVENGAYKAQGVQLTAKLGYPITDDLDIYTRLGGMVW
RADTKSNVYGKNHDTGVSPVFAGGVEYAITPEIATRLEYQWTNNIGDAHTIGTRPDNGMLSLGV
SYRFGQGEAAPVVAPAPAPAPEVQTKHFTLKSDVLFNFNKATLKPEGQAALDQLYSQLSNLDPK
DGSVVVLGYTDRIGSDAYNQGLSERRAQSVVDYLISKGIPADKISARGMGESNPVTGNTCDNVK
QRAALIDCLAPDRRVEIEVKGIKDVVTQPQA

>seq204
MKKTAIAIAVALAGFATVAQAAPKDNTWYTGAKLGWSQYHDTGFINNNGPTHENQLGAGAFG
GYQVNPYVGFEMGYDWLGRMPYKGSVENGAYKAQGVQLTAKLGYPITDDLDIYTRLGGMVW
RADTKSNVYGKNHDTGVSPVFAGGVEYAITPEIATRLEYQWTNNIGDAHTIGTRPDNGMLSLGV
SYRFGQGEAAPVVAPAPAPAPEVQTKHFTLKSDVLFNFNKATLKPEGQAALDQLYSQLSNLDPK
DGSVVVLGYTDRIGSDAYNQGLSERRAQSVVDYLISKGIPADKISARGMGESNPVTGNTCDNVK
QRAALIDCLAPDRRVEIEVKGIKDVVTQPQA

>seq205
MKKTAIAIAVALAGFATVAQAAPKDNTWYTGAKLGWSQYHDTGFIDNNGPTHENQLGAGAFG
GYQVNPYVGFEMGYDWLGRMPYKGSVENGAYKAQGVQLTAKLGYPITDDLDVYTRLGGMV
WRADTKAHNNVTGESEKNHDTGVSPVFAGGVEWAITPEIATRLEYQWTNNIGDAHTIGTRPDN
GLLSLGVSYRFGQGEAAPVVAPAPAPAPEVQTKHFTLKSDVLFNFNKATLKPEGQAALDQLYSQ
LSNLDPKDGSVVVLGYTDRIGSDAYNQGLSERRAQSVVDYLISKGIPADKISARGMGESNPVTG
NTCDNVKQRAALIDCLAPDRRVEIEVKGIKDVVTQPQA

>seq206
MKKTAIAIAVALAGFATVAQAAPKDNTWYTGAKLGWSQYHDTGFIPNNGPTHENQLGAGAFG
GYQVNPYVGFEMGYDWLGRMPYKGDNINGAYKAQGVQLTAKLGYPITDDLDVYTRLGGMVW
RADTKANVPGGASFKDHDTGVSPVFAGGVEYAITPEIATRLEYQWTNNIGDAHTIGTRPDNGLL
SLGVSYRFGQGEAAPVVAPAPAPAPEVQTKHFTLKSDVLFNFNKATLKPEGQAALDQLYSQLSN
LDPKDGSVVVLGYTDRIGSDAYNQGLSERRAQSVVDYLISKGIPADKISARGMGESNPVTGNTC
DNVKQRAALIDCLAPDRRVEIEVKGIKDVVTQPQA

>seq207
MKKTAIAIAVALAGFATVAQAAPKDNTWYTGAKLGWSQYHDTGFIPNNGPTHENQLGAGAFG
GYQVNPYVGFEMGYDWLGRMPYKGDNINGAYKAQGVQLTAKLGYPITDDLDVYTRLGGMVW
RADTKANVPGGASFKDHDTGVSPVFAGGVEYAITPEIATRLEYQWTNNIGDANTIGTRPDNGLL
SLGVSYRFGQGEAAPVVAPAPAPAPEVQTKHFTLKSDVLFNFNKATLKPEGQAALDQLYSQLSN

LDPKDGSVVVLGYTDRIGSDAYNQGLSERRAQSVVDYLISKGIPADKISARGMGESNPVTGNTC
DNVKQRAALIDCLAPDRRVEIEVKGIKDVVTQPQA

>seq211
MKKTAIAIAVALAGFATVAQAAPKDNTWYTGAKLGWSQYHDTGFINNNGPTHENQLGAGAFG
GYQVNPYVGFEMGYDWLGRMPYKGSVENGAYKAQGVQLTAKLGYPITDDLDIYTRLGGMVW
RADTKSNVYGKNHDTGVSPVFAGGVEYAITPEIATRLEYQWTNNIGDAHTIGTRPDNGMLSLGV
SYRFGQGEAAPVVAPAPAPAPEVQTKHFTLKSDVLFNFNKATLKPEGQAALDQLYSQLSNLDPK
DGSVVVLGYTDRIGSDAYNQGLSERRAQSVVDYLISKGIPADKISARGMGESNPVTGNTCDNVK
QRAALIDCLAPDRRVEIEVKGIKDVVTQPQA

>seq217
MKKTAIAIAVALAGFATVAQAAPKDNTWYTGAKLGWSQYHDTGFIPNNGPTHENQLGAGAFG
GYQVNPYVGFEMGYDWLGRMPYKGDNINGAYKAQGVQLTAKLGYPITDDLDVYTRLGGMVW
RADTKANVPGGASFKDHDTGVSPVFAGGVEYAITPEIATRLEYQWTNNIGDANTIGTRPDNGLL
SLGVSYRFGQGEAAPVVAPAPAPAPEVQTKHFTLKSDVLFNFNKATLKPEGQAALDQLYSQLSN
LDPKDGSVVVLGYTDRIGSDAYNQGLSERRAQSVVDYLISKGIPADKISARGMGESNPVTGNTC
DNVKQRAALIDCLAPDRRVEIEVKGIKDVVTQPQA

>seq222
MKKTAIAIAVALAGFATVAQAAPKDNTWYTGAKLGWSQYHDTGFIPNNGPTHENQLGAGAFG
GYQVNPYVGFEMGYDWLGRMPYKGDNINGAYKAQGVQLTAKLGYPITDDLDIYTRLGGMVW
RADTKANVPGGASFKDHDTGVSPVFAGGVEYAITPEIATRLEYQWTNNIGDANTIGTRPDNGLL
SLGVSYRFGQGEAAPVVAPAPAPAPEVQTKHFTLKSDVLFNFNKATLKPEGQAALDQLYSQLSN
LDPKDGSVVVLGYTDRIGSDAYNQGLSERRAQSVVDYLISKGIPADKISARGMGESNPVTGNTC
DNVKQRAALIDCLAPDRRVEIEVKGIKDVVTQPQA

>seq226
MKKTAIAIAVALAGFATVAQAAPKDNTWYTGAKLGWSQYHDTGFINNNGPTHENQLGAGAFG
GYQVNPYVGFEMGYDWLGRMPYKGSVENGAYKAQGVQLTAKLGYPITDDLDIYTRLGGMVW
RADTKSNVYGKNHDTGVSPVFAGGVEYAITPEIATRLEYQWTNNIGDAHTIGTRPDNGMLSLGV
SYRFGQGEAAPVVAPAPAPAPEVQTKHFTLKSDVLFNFNKATLKPEGQAALDQLYSQLSNLDPK
DGSVVVLGYTDRIGSDAYNQGLSERRAQSVVDYLISKGIPADKISARGMGESNPVTGNTCDNVK
QRAALIDCLAPDRRVEIEVKGIKDVVTQPQA

>seq230
MKKTAIAIAVALAGFATVAQAAPKDNTWYTGAKLGWSQYHDTGFINNNGPTHENQLGAGAFG
GYQVNPYVGFEMGYDWLGRMPYKGSVENGAYKAQGVQLTAKLGYPITDDLDIYTRLGGMVW
RADTKSNVYGKNHDTGVSPVFAGGVEYAITPEIATRLEYQWTNNIGDAHTIGTRPDNGMLSLGV
SYRFGQGEAAPVVAPAPAPAPEVQTKHFTLKSDVLFNFNKATLKPEGQAALDQLYSQLSNLDPK
DGSVVVLGYTDRIGSDAYNQGLSERRAQSVVDYLISKGIPADKISARGMGESNPVTGNTCDNVK
QRAALIDCLAPDRRVEIEVKGIKDVVTQPQA

>seq236
MKKTAIAIAVALAGFATVAQAAPKDNTWYTGAKLGWSQYHDTGFIPNNGPTHENQLGAGAFG
GYQVNPYVGFEMGYDWLGRMPYKGDNINGAYKAQGVQLTAKLGYPITDDLDVYTRLGGMVW
RADTKANVPGGASFKDHDTGVSPVFAGGVEYAITPEIATRLEYQWTNNIGDANTIGTRPDNGLL
SLGVSYRFGQGEAAPVVAPAPAPAPEVQTKHFTLKSDVLFNFNKATLKPEGQAALDQLYSQLSN
LDPKDGSVVVLGYTDRIGSDAYNQGLSERRAQSVVDYLISKGIPADKISARGMGESNPVTGNTC
DNVKQRAALIDCLAPDRRVEIEVKGIKDVVTQPQA

>seq248
MKKTAIAIAVALAGFATVAQAAPKDNTWYTGAKLGWSQYHDTGFIPNNGPTHENQLGAGAFG
GYQVNPYVGFEMGYDWLGRMPYKGDNINGAYKAQGVQLTAKLGYPITDDLDVYTRLGGMVW
RADTKANVPGGASFKDHDTGVSPVFAGGVEYAITPEIATRLEYQWTNNIGDANTIGTRPDNGLL
SLGVSYRFGQGEAAPVVAPAPAPAPEVQTKHFTLKSDVLFNFNKATLKPEGQAALDQLYSQLSN
LDPKDGSVVVLGYTDRIGSDAYNQGLSERRAQSVVDYLISKGIPADKISARGMGESNPVTGNTC
DNVKQRAALIDCLAPDRRVEIEVKGIKDVVTQPQA

>seq249
MKKTAIAIAVALAGFATVAQAAPKDNTWYTGAKLGWSQYHDTGFIDNNGPTHENQLGAGAFG
GYQVNPYVGFEMGYDWLGRMPYKGDNINGAYKAQGVQLTAKLGYPITDDLDVYTRLGGMVW
RADTKSNVPGGVSTKDHDTGVSPVFAGGVEYAITPEIATRLEYQWTNNIGDAHTIGTRPDNGML
SLGVSYRFGQGEAAPVVAPAPAPAPEVQTKHFTLKSDVLFNFNKATLKPEGQAALDQLYSQLSN
LDPKDGSVVVLGYTDRIGSDAYNQGLSERRAQSVVDYLISKGIPADKISARGMGESNPVTGNTC
DNVKQRAALIDCLAPDRRVEIEVKGIKDVVTQPQA

>seq256
MKKTAIAIAVALAGFATVAQAAPKDNTWYTGAKLGWSQYHDTGFIPNNGPTHENQLGAGAFG
GYQVNPYVGFEMGYDWLGRMPYKGDNINGAYKAQGVQLTAKLGYPITDDLDVYTRLGGMVW
RADTKSNVPGGASTKDHDTGVSPVFAGGVEYAITPEIATRLEYQWTNNIGDAHTIGTRPDNGML
SLGVSYRFGQGEAAPVVAPAPAPAPEVQTKHFTLKSDVLFTFNKATLKPEGQAALDQLYSQLSN
LDPKDGSVVVLGYTDRIGSDAYNQALSERRAQSVVDYLISKGIPADKISARGMGESNPVTGNTC
DNVKQRAALIDCLAPDRRVEIEVKGIKDVVTQPQA

>seq257
MKKTAIAIAVALAGFATVAQAAPKDNTWYTGAKLGWSQYHDTGFIPNNGPTHENQLGAGAFG
GYQVNPYVGFEMGYDWLGRMPYKGDNINGAYKAQGVQLTAKLGYPITDDLDVYTRLGGMVW
RADTKANVPGGASFKDHDTGVSPVFAGGVEYAITPEIATRLEYQWTNNIGDANTIGTRPDNGLL
SLGVSYRFGQGEAAPVVAPAPAPAPEVQTKHFTLKSDVLFNFNKATLKPEGQAALDQLYSQLSN
LDPKDGSVVVLGYTDRIGSDAYNQGLSERRAQSVVDYLISKGIPADKISARGMGESNPVTGNTC
DNVKQRAALIDCLAPDRRVEIEVKGIKDVVTQPQA

>seq270
MKKTAIAIAVALAGFATVAQAAPKDNTWYTGAKLGWSQYHDTGFINNNGPTHENQLGAGAFG
GYQVNPYVGFEMGYDWLGRMPYKGSVENGAYKAQGVQLTAKLGYPITDDLDIYTRLGGMVW
RADTKSNVYGKNHDTGVSPVFAGGVEYAITPEIATRLEYQWTNNIGDAHTIGTRPDNGMLSLGV
SYRFGQGEAAPVVAPAPAPAPEVQTKHFTLKSDVLFNFNKATLKPEGQAALDQLYSQLSNLDPK
DGSVVVLGYTDRIGSDAYNQGLSERRAQSVVDYLISKGIPADKISARGMGESNPVTGNTCDNVK
QRAALIDCLAPDRRVEIEVKGIKDVVTQPQA

>seq280
MKKTAIAIAVALAGFATVAQAAPKDNTWYTGAKLGWSQYHDTGFIPNNGPTHENQLGAGAFG
GYQVNPYVGFEMGYDWLGRMPYKGDNINGAYKAQGVQLTAKLGYPITDDLDVYTRLGGMVW
RADTKANVPGGASFKDHDTGVSPVFAGGVEYAITPEIATRLEYQWTNNIGDANTIGTRPDNGLL
SLGVSYRFGQGEAAPVVAPAPAPAPEVQTKHFTLKSDVLFNFNKATLKPEGQAALDQLYSQLSN
LDPKDGSVVVLGYTDRIGSDAYNQGLSERRAQSVVDYLISKGIPADKISARGMGESNPVTGNTC
DNVKQRAALIDCLAPDRRVEIEVKGIKDVVTQPQA

>seq281
MKKTAIAIAVALAGFATVAQAAPKDNTWYTGAKLGWSQYHDTGFIPNNGPTHENQLGAGAFG
GYQVNPYVGFEMGYDWLGRMPYKGDNINGAYKAQGVQLTAKLGYPITDDLDIYTRLGGMVW

RADTKANVPGGASFKDHDTGVSPVFAGGVEYAITPEIATRLEYQWTNNIGDAHTIGTRPDNGM
LSLGVSYRFGQGEAAPVVAPAPAPAPEVQTKHFTLKSDVLFNFNKATLKPEGQAALDQLYSQLS
NLDPKDGSVVVLGYTDRIGSDAYNQGLSERRAQSVVDYLISKGIPADKISARGMGESNPVTGNT
CDNVKQRAALIDCLAPDRRVEIEVKGIKDVVTQPQA

>seq284
MKKTAIAIAVALAGFATVAQAAPKDNTWYTGAKLGWSQYHDTGFINNNGPTHENQLGAGAFG
GYQVNPYVGFEMGYDWLGRMPYKGSVENGAYKAQGVQLTAKLGYPITDDLDIYTRLGGMVW
RADTKSNVYGKNHDTGVSPVFAGGVEYAITPEIATRLEYQWTNNIGDAHTIGTRPDNGMLSLGV
SYRFGQGEAAPVVAPAPAPAPEVQTKHFTLKSDVLFNFNKATLKPEGQAALDQLYSQLSNLDPK
DGSVVVLGYTDRIGSDAYNQGLSERRAQSVVDYLISKGIPADKISARGMGESNPVTGNTCDNVK
QRAALIDCLAPDRRVEIEVKGIKDVVTQPQA

>seq286
MKKTAIAIAVALAGFATVAQAAPKDNTWYTGAKLGWSQYHDTGFINNNGPTHENQLGAGAFG
GYQVNPYVGFEMGYDWLGRMPYKGSVENGAYKAQGVQLTAKLGYPITDDLDIYTRLGGMVW
RADTKSNVYGKNHDTGVSPVFAGGVEYAITPEIATRLEYQWTNNIGDAHTIGTRPDNGMLSLGV
SYRFGQGEAAPVVAPAPAPAPEVQTKHFTLKSDVLFNFNKATLKPEGQAALDQLYSQLSNLDPK
DGSVVVLGYTDRIGSDAYNQGLSERRAQSVVDYLISKGIPADKISARGMGESNPVTGNTCDNVK
QRAALIDCLAPDRRVEIEVKGIKDVVTQPQA

>seq289
MKKTAIAIAVALAGFATVAQAAPKDNTWYTGAKLGWSQYHDTGFINNNGPTHENQLGAGAFG
GYQVNPYVGFEMGYDWLGRMPYKGSVENGAYKAQGVQLTAKLGYPITDDLDIYTRLGGMVW
RADTKSNVYGKNHDTGVSPVFAGGVEYAITPEIATRLEYQWTNNIGDAHTIGTRPDNGMLSLGV
SYRFGQGEAAPVVAPAPAPAPEVQTKHFTLKSDVLFNFNKATLKPEGQAALDQLYSQLSNLDPK
DGSVVVLGYTDRIGSDAYNQGLSERRAQSVVDYLISKGIPADKISARGMGESNPVTGNTCDNVK
QRAALIDCLAPDRRVEIEVKGIKDVVTQPQA

>seq292
MKKTAIAIAVALAGFATVAQAAPKDNTWYTGAKLGWSQYHDTGFINNNGPTHENQLGAGAFG
GYQVNPYVGFEMGYDWLGRMPYKGSVENGAYKAQGVQLTAKLGYPITDDLDIYTRLGGMVW
RADTKSNVYGKNHDTGVSPVFAGGVEYAITPEIATRLEYQWTNNIGDAHTIGTRPDNGMLSLGV
SYRFGQGEAAPVVAPAPAPAPEVQTKHFTLKSDVLFNFNKATLKPEGQAALDQLYSQLSNLDPK
DGSVVVLGYTDRIGSDAYNQGLSERRAQSVVDYLISKGIPADKISARGMGESNPVTGNTCDNVK
QRAALIDCLAPDRRVEIEVKGIKDVVTQPQA

>seq298
MKKTAIAIAVALAGFATVAQAAPKDNTWYTGAKLGWSQYHDTGFIPNNGPTHENQLGAGAFG
GYQVNPYVGFEMGYDWLGRMPYKGDNINGAYKAQGVQLTAKLGYPITDDLDVYTRLGGMVW
RADTKANVPGGASFKDHDTGVSPVFAGGVEYAITPEIATRLEYQWTNNIGDANTIGTRPDNGLL
SLGVSYRFGQGEAAPVVAPAPAPAPEVQTKHFTLKSDVLFNFNKATLKPEGQAALDQLYSQLSN
LDPKDGSVVVLGYTDRIGSDAYNQGLSERRAQSVVDYLISKGIPADKISARGMGESNPVTGNTC
DNVKQRAALIDCLAPDRRVEIEVKGIKDVVTQPQA

>seq307
MKKTAIAIAVALAGFATVAQAAPKDNTWYTGAKLGWSQYHDTGFINNNGPTHENQLGAGAFG
GYQVNPYVGFEMGYDWLGRMPYKGDNINGAYKAQGVQLTAKLGYPITDDLDIYTRLGGMVW
RADTKANVPGGASFKDHDTGVSPVFAGGVEYAITPEIATRLEYQWTNNIGDAHTIGTRPDNGLL
SLGVSYRFGQGEAAPVVAPAPAPAPEVQTKHFTLKSDVLFNFNKATLKPEGQAALDQLYSQLSN

LDPKDGSVVVLGYTDRIGSDAYNQ**G**LSERRAQSVVDYLISKGIP**A**DKISARGMGESNPVTGNTC
DNVKQRAALIDCLAPDRRVEIEVKGIKDVVTQPQA

>seq308
MKKTAIAIAVALAGFATVAQAAPKDNTWYTGAKLGWSQYHDTGFI**D**NNGPTHENQLGAGAFG
GYQVNPYVGFEMGYDWLGRMPYKG**SVE**NGAYKAQGVQLTAKLGYPITDDLD**V**YTRLGGMV
WRADTK**AHNNVTGESE**K**N**HDTGVSPVFAGGVE**W**AITPEIATRLEYQWTNNIGDA**H**TIGTRPDN
G**L**LSLGVSYRFGQGE**A**APVV**A**PAPAPAPEVQTKHFTLKSDVLF**N**FNKATLKPEGQAALDQLYSQ
LSNLDPKDGSVVVLGYTDRIGSDAYNQ**G**LSERRAQSVVDYLISKGIP**A**DKISARGMGESNPVTG
NTCDNVKQRAALIDCLAPDRRVEIEVKGIKDVVTQPQA

>seq313
MKKTAIAIAVALAGFATVAQAAPKDNTWYTGAKLGWSQYHDTGFI**N**NNGPTHENQLGAGAFG
GYQVNPYVGFEMGYDWLGRMPYKG**SVE**NGAYKAQGVQLTAKLGYPITDDLD**I**YTRLGGMVW
RADTK**SNVYG**K**N**HDTGVSPVFAGGVE**Y**AITPEIATRLEYQWTNNIGDA**H**TIGTRPDNG**M**LSLGV
SYRFGQGE**A**APVV**A**PAPAPAPEVQTKHFTLKSDVLF**N**FNKATLKPEGQAALDQLYSQLSNLDPK
DGSVVVLGYTDRIGSDAYNQ**G**LSERRAQSVVDYLISKGIP**A**DKISARGMGESNPVTGNTCDNVK
QRAALIDCLAPDRRVEIEVKGIKDVVTQPQA

>seq315
MKKTAIAIAVALAGFATVAQAAPKDNTWYTGAKLGWSQYHDTGFI**N**NNGPTHENQLGAGAFG
GYQVNPYVGFEMGYDWLGRMPYKG**SVE**NGAYKAQGVQLTAKLGYPITDDLD**I**YTRLGGMVW
RADTK**SNVYG**K**N**HDTGVSPVFAGGVE**Y**AITPEIATRLEYQWTNNIGDA**H**TIGTRPDNG**M**LSLGV
SYRFGQGE**A**APVV**A**PAPAPAPEVQTKHFTLKSDVLF**N**FNKATLKPEGQAALDQLYSQLSNLDPK
DGSVVVLGYTDRIGSDAYNQ**G**LSERRAQSVVDYLISKGIP**A**DKISARGMGESNPVTGNTCDNVK
QRAALIDCLAPDRRVEIEVKGIKDVVTQPQA

>seq329
MKKTAIAIAVALAGFATVAQAAPKDNTWYTGAKLGWSQYHDTGFI**P**NNGPTHENQLGAGAFG
GYQVNPYVGFEMGYDWLGRMPYKG**DNI**NGAYKAQGVQLTAKLGYPITDDLD**V**YTRLGGMVW
RADTK**ANVPGGASF**K**D**HDTGVSPVFAGGVE**Y**AITPEIATRLEYQWTNNIGDA**N**TIGTRPDNG**L**L
SLGVSYRFGQGE**A**APVV**A**PAPAPAPEVQTKHFTLKSDVLF**N**FNKATLKPEGQAALDQLYSQLSN
LDPKDGSVVVLGYTDRIGSDAYNQ**G**LSERRAQSVVDYLISKGIP**A**DKISARGMGESNPVTGNTC
DNVKQRAALIDCLAPDRRVEIEVKGIKDVVTQPQA

>seq331
MKKTAIAIAVALAGFATVAQAAPKDNTWYTGAKLGWSQYHDTGFI**N**NNGPTHENQLGAGAFG
GYQVNPYVGFEMGYDWLGRMPYKG**SVE**NGAYKAQGVQLTAKLGYPITDDLD**I**YTRLGGMVW
RADTK**SNVYG**K**N**HDTGVSPVFAGGVE**Y**AITPEIATRLEYQWTNNIGDA**H**TIGTRPDNG**M**LSLGV
SYRFGQGE**A**APVV**A**PAPAPAPEVQTKHFTLKSDVLF**N**FNKATLKPEGQAALDQLYSQLSNLDPK
DGSVVVLGYTDRIGSDAYNQ**G**LSERRAQSVVDYLISKGIP**A**DKISARGMGESNPVTGNTCDNVK
QRAALIDCLAPDRRVEIEVKGIKDVVTQPQ

>seq333
MKKTAIAIAVALAGFATVAQAAPKDNTWYTGAKLGWSQYHDTGFI**D**NNGPTHENQLGAGAFG
GYQVNPYVGFEMGYDWLGRMPYKG**DNI**NGAYKAQGVQLTAKLGYPITDDLD**I**YTRLGGMVW
RADTK**ANVPGGASF**K**D**HDTGVSPVFAGGVE**Y**AITPEIATRLEYQWTNNIGDA**H**TIGTRPDNG**L**L
SLGVSYRFGQGE**A**APVV**A**PAPAPAPEVQTKHFTLKSDVLF**N**FNKATLKPEGQAALDQLYSQLSN
LDPKDGSVVVLGYTDRIGSDAYNQ**G**LSERRAQSVVDYLISKGIP**A**DKISARGMGESNPVTGNTC
DNVKQRAALIDCLAPDRRVEIEVKGIKDVVTQPQA

>seq342
MKKTAIAIAVALAGFATVAQAAPKDNTWYTGAKLGWSQYHDTGFI**D**NNGPTHENQLGAGAFG
GYQVNPYVGFEMGYDWLGRMPYKG**SVE**NGAYKAQGVQLTAKLGYPITDDLD**V**YTRLGGMV
WRADTK**AHNNVTGESE**K**N**HDTGVSPVFAGGVE**W**AITPEIATRLEYQWTNNIGDA**H**TIGTRPDN
G**L**LSLGVSYRFGQGE**A**APVV**A**PAPAPAPEVQTKHFTLKSDVLF**N**FNKATLKPEGQAALDQLYSQ
LSNLDPKDGSVVVLGYTDRIGSDAYNQ**G**LSERRAQSVVDYLISKGIP**A**DKISARGMGESNPVTG
NTCDNVKQRAALIDCLAPDRRVEIEVKGIKDVVTQPQA

>seq385
MKKTAIAIAVALAGFATVAQAAPKDNTWYTGAKLGWSQYHDTGFI**P**NNGPTHENQLGAGAFG
GYQVNPYVGFEMGYDWLGRMPYKG**DNI**NGAYKAQGVQLTAKLGYPITDDLD**I**YTRLGGMVW
RADTK**ANVPGGASY**K**D**HDTGVSPVFAGGVE**Y**AITPEIATRLEYQWTNNIGDA**H**TIGTRPDNG**L**L
SLGVSYRFGQGE**A**APVV**A**PAPAPAPEVQTKHFTLKSDVLF**N**FNKATLKPEGQAALDQLYSQLSN
LDPKDGSVVVLGYTDRIGSDAYNQ**G**LSERRAQSVVDYLISKGIP**S**DKISARGMGESNPVTGNTCD
NVKQRAALIDCLAPDRRVEIEVKGIKDVVTQPQA

>seq391
MKKTAIAIAVALAGFATVAQAAPKDNTWYTGAKLGWSQYHDTGFI**P**NNGPTHENQLGAGAFG
GYQVNPYVGFEMGYDWLGRMPYKG**DNI**NGAYKAQGVQLTAKLGYPITDDLD**V**YTRLGGMVW
RADTK**ANVPGGASF**K**D**HDTGVSPVFAGGVE**Y**AITPEIATRLEYQWTNNIGDA**H**TIGTRPDNG**M**
LSLGVSYRFGQGE**A**APVV**A**PAPAPAPEVQTKHFTLKSDVLF**N**FNKATLKPEGQAALDQLYSQLS
NLDPKDGSVVVLGYTDRIGSDAYNQ**G**LSERRAQSVVDYLISKGIP**A**DKISARGMGESNPVTGNT
CDNVKQRAALIDCLAPDRRVEIEVKGIKDVVTQPQA
