# Supplementary material for: Allelic Variation in Outer Membrane Protein A and Its Influence on Attachment of Escherichia coli to Corn Stover
Source: Front Microbiol. 2017 May 3;8:708. doi: 10.3389/fmicb.2017.00708 (PMC5413513; doi:10.3389/fmicb.2017.00708)

FIGURE S1 Motility agar stab test confirmed the removal of *fliC* gene from genome of strain 44, 117 and MG1655.

Strain: 44 44:Δ*fliC* 117 117:Δ*fliC* MG1655 MG1655:Δ*fliC*

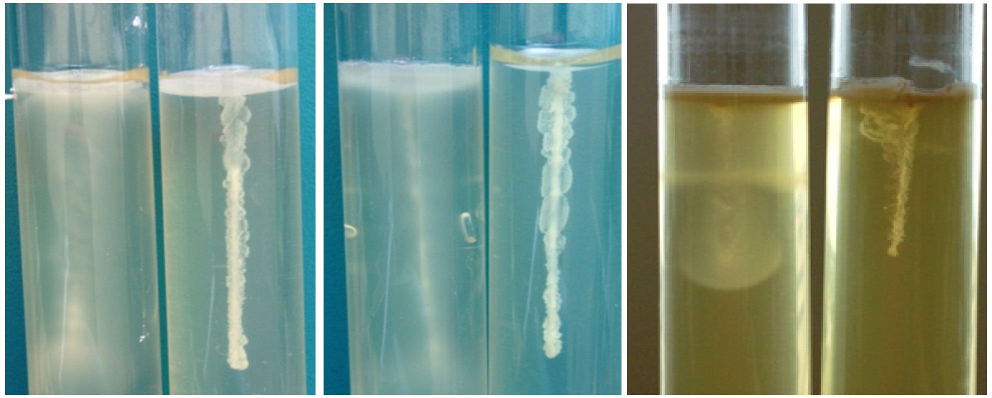

Supplement: Supplementary file 5 [file Image1.PDF]
