# Supplementary material for: Allelic Variation in Outer Membrane Protein A and Its Influence on Attachment of Escherichia coli to Corn Stover
Source: Front Microbiol. 2017 May 3;8:708. doi: 10.3389/fmicb.2017.00708 (PMC5413513; doi:10.3389/fmicb.2017.00708)

FIGURE S2 Flagella of *E. coli* cells were removed by blending. Transmission electron microscopy (TEM) images of *E. coli* MG1655 cells before blending (A and B), and after blending (C and D) show that flagella were removed.

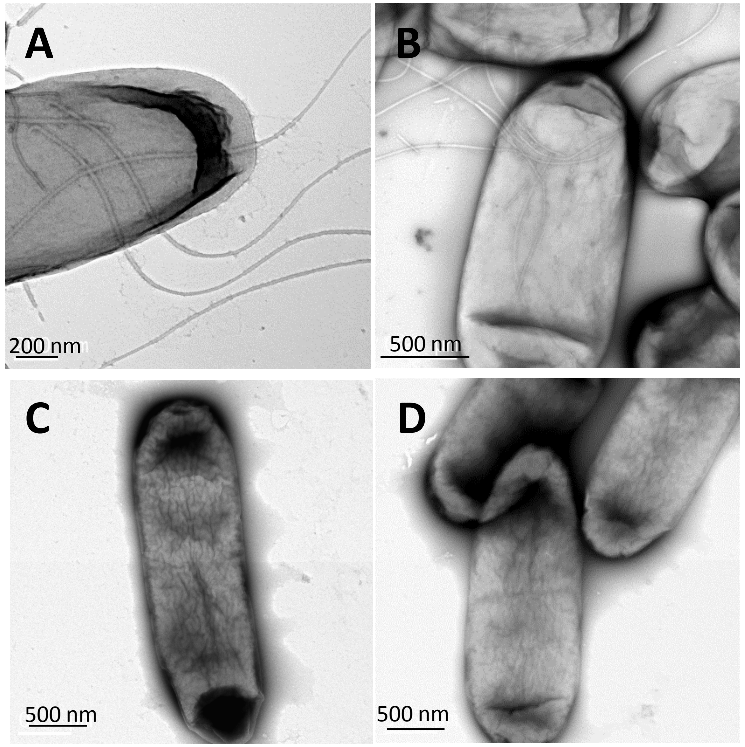

Supplement: Supplementary file 6 [file Image2.PDF]
